# Exploration of compounds to inhibit the Panton-Valentine leukocidin of *Staphylococcus aureus*

**DOI:** 10.1007/s00430-024-00803-1

**Published:** 2024-09-19

**Authors:** Tobias Grebe, Mithra Tatjana Sarkari, Angelika Cherkaoui, Frieder Schaumburg

**Affiliations:** Institute of Medical Microbiology, University of Münster, Domagkstraße 10, 48149 Münster, Germany

**Keywords:** *Staphylococcus aureus*, Panton-Valentine leukocidin, Complement 5a receptor, CD88, CD45, Antagonist

## Abstract

The Panton-Valentine leukocidin (PVL) of *Staphylococcus aureus* is associated with necrotizing infections. After binding to complement 5a receptor (C5aR/CD88) and CD45 it causes cytolysis in polymorphonuclear neutrophils (PMNs) as well as inflammasome activation in monocytes. The objective of this study was to test if (ant)agonists of C5aR and CD45 can attenuate the effect of PVL on PMNs and monocytes. We tested the effect of various concentrations of six C5aR (ant)agonists (avacopan, BM213, DF2593A, JPE-1375, PMX205 and W-54011) and one CD45 antagonist (NQ301) to attenuate the cytotoxic effect of PVL on human PMNs and monocytes in vitro. Shifts in the half-maximal effective concentration (EC_50_) of PVL to achieve a cytotoxic effect on PMNs and modulation of inflammatory cytokine response from monocytes were determined by flow cytometry and IL-1β detection. Pre-treatment of PMNs with avacopan, PMX205 and W-54,011 resulted in 3.6- to 4.3-fold shifts in the EC_50_ for PVL and were able to suppress IL-1β secretion by human monocytes in the presence of PVL. BM213, DF2593A and NQ301 were unable to change the susceptibility of PMNs towards PVL or reduce inflammasome activation in monocytes. Avacopan, PMX205 and W-54,011 showed protection against PVL-induced cytotoxicity and suppressed IL-1β secretion by monocytes. Clinical studies are needed to prove whether these substances can be used therapeutically as repurposed drugs.

**Supplementary Information:**

The online version contains supplementary material available at 10.1007/s00430-024-00803-1.

## Introduction

The *Staphylococcus aureus* Panton-Valentine leukocidin (PVL) is a bi-component pore-forming toxin predominantly associated with severe skin and soft tissue infections (SSTI), such as pyomyositis, and is believed to contribute to the development of necrotizing pneumonia [1, 2]. Following secretion, the two components of PVL, LukS-PV and LukF-PV, bind to the complement 5a receptor (C5aR/CD88) and CD45, respectively. These receptors are expressed on polymorphonuclear leukocytes (PMNs, i.e. neutrophil granulocytes), macrophages, and monocytes. After hetero-oligomerization, PVL is integrated into the membrane as an octameric pore eventually leading to cytolysis [3, 4]. Binding of PVL to monocytes induces inflammasome activation and subsequent release of pro-inflammatory cytokines [5].

In the quest to combat infections, there is an increasing emphasis on exploring virulence-modifying strategies to complement antibiotic therapy. As an example, anakinra, an IL-1 receptor antagonist, has the potential to alleviate the severity of PVL-mediated necrotizing pneumonia [6]. Since competition with the cellular receptors of PVL interferes with PVL toxicity [4, 7], this study aimed to explore inhibitors of PVL using human leukocyte-based assays to evaluate the efficacy of C5aR and CD45 (ant)agonists in attenuating PVL-mediated cytotoxicity on PMNs and inflammasome activation in monocytes.

## Materials and methods

### PVL inhibitors

We selected various compounds known to interact with C5aR or CD45, the cellular targets of PVL. These compounds were avacopan/CCX168 (C5aR antagonist, Thermo Fisher Scientific, Waltham, MA, USA), BM213 (C5aR agonist, MedChemExpress, Monmouth Junction, NJ, USA), DF2593A (C5aR antagonist, Sigma-Aldrich, St. Louis, MO, USA), JPE-1375 (C5aR antagonist, MedChemExpress), PMX205 (C5aR antagonist, Hycultec, Beutelsbach, Germany), W-54,011 (C5aR antagonist, MedChemExpress) as well as NQ301 (CD45 inhibitor, MedChemExpress). Avacopan, DF2593A, JPE-1375, PMX205, W-54,011, and NQ301 were reconstituted in DMSO, BM213 was dissolved in PBS, and stocks were stored at -20 °C. Working solutions were freshly prepared by serially diluting in PBS.

### Production and purification of PVL

The LukS-PV and LukF-PV PVL subunits were recombinantly expressed in *Escherichia coli* TG1 using the IPTG-inducible pQE30UA vector as previously reported [8]. Briefly, polyhistidine-tagged proteins were affinity-purified from cell lysates on Ni-nitrilotriacetic acid columns (Qiagen, Hilden, Germany). Subsequently, buffer exchange with phosphate-buffered saline (PBS) was performed on PD-10 Sephadex G-25 medium columns (Cytiva, Marlborough, USA).

### Isolation of human leukocytes

Human PMNs and monocytes were freshly isolated from blood of healthy volunteers [9]. Following dextran sedimentation, cells were separated by density gradient centrifugation using Ficoll-Paque Plus (Sigma-Aldrich) following the manufacturer’s instructions. Remaining erythrocytes in the pellet with PMNs were removed by hypotonic lysis in sterile distilled water. Monocytes in the layer of peripheral blood mononuclear cells were selected by adhesion to tissue culture plastic (Corning Costar, Thermo Fisher Scientific). Finally, PMNs and monocytes were suspended in RPMI-1640 medium (Sigma-Aldrich) supplemented with 5% fetal bovine serum (Sigma-Aldrich) at a final concentration of 1 × 10^6^ and 5 × 10^6^ cells/ml, respectively. Cell viability was determined to be greater than 95%, as assessed by flow cytometry.

### PMN competition assay

PMNs were pre-treated with inhibitory compounds (1 nM, 10 nM, 100 nM, 1 µM, 10µM) for 15 min at room temperature, unless otherwise stated. Subsequently, various equimolar concentrations of recombinant LukS-PV and LukF-PV (0.125-8 nM) were added for incubation (1 h, room temperature, 5 rpm). After staining cells with 5 µg/ml propidium iodide (PI, Sigma-Aldrich), PVL-induced cell damage was analyzed using an Accuri C6 Flow cytometer (BD Biosciences, Heidelberg, Germany). For each tested competitor concentration, the EC_50_ of PVL was determined as the concentration of PVL resulting in 50% PI-positive cells. Controls included addition of competitors without addition of PVL and addition of 0.1% DMSO (corresponding to highest diluent concentration of compounds) to PVL.

### Inflammasome activation

Monocytes were isolated from venous blood and allowed to recover overnight (37 °C, 5% CO_2_) at a concentration of 5 × 10^6^ cells/well in a 12-well plate (Corning Costar). On the following day, monocytes were pre-incubated for 15 min with competitors before treatment with 0.25 and 0.5 nM PVL (3 h, 37 °C, 5% CO_2_). Cell-free supernatants were stored at -20 °C and IL-1β was quantified by ELISA (Thermo Fisher Scientific) to assess inflammasome activation. Monocytes were detached with 2.5 mM EDTA in PBS (4 °C, 30 min), stained with 5 µg/ml PI and the proportion of PI-positive monocytes was measured using an Accuri C6 Flow cytometer (BD Biosciences).

### Statistical analysis

All statistical analyzes were performed using Prism 10 (GraphPad Software, San Diego, USA). Dose-response curves were fitted using asymmetric five-parameter nonlinear regression to calculate EC_50_ values of PVL. Differences between fitted curves and EC_50_ values for tested concentrations of competitors were calculated using two-way ANOVA with Dunnet’s *post hoc* test for multiple comparisons.

## Results

### Compounds attenuate cytotoxicity of PVL on PMNs

We measured the half-maximal effective concentration (EC_50_) of various compounds to attenuate the effect of PVL on PMNs (i.e. cytotoxicity) and monocytes (i.e. inflammasome activation).

**Fig. 1:**
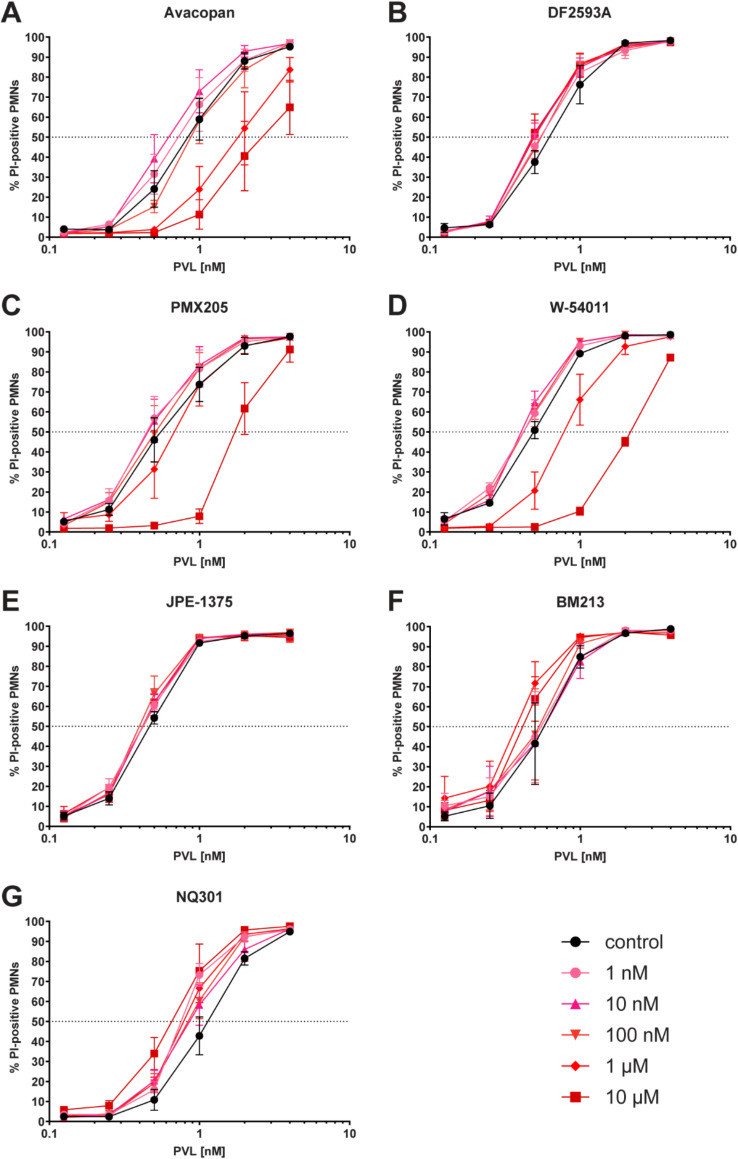
Inhibitory effect of C5a or CD45 receptor (ant)agonists on Panton-Valentine leukocidin (PVL)-induced cytotoxicity in polymorphonuclear neutrophils (PMNs) Inhibitor concentrations ranged from 1 nM to 10 µM. C5a receptor antagonists avacopan (**A**), DF2593A (**B**), PMX2015 (**C**), W-54,011 (**D**), JPE-1375 (**E**), C5a receptor agonist BM213 (**F**), and CD45 antagonist NQ301 (**G**) were added to PMNs prior to incubation with PVL (0.125-4 nM). The cytotoxic effect of PVL is displayed as mean percentage ± SEM of propidium iodide (PI)-stained PMNs from three independent experiments quantified by flow cytometry

Avacopan, PMX205 and W-54,011 changed the EC_50_ of PVL towards human PMNs markedly. A significant shift compared to controls was achieved at ≥ 1 µM for avacopan and ≥ 10 µM for PMX205 and W-54,011 (Fig. 1; Table 1). In contrast, the cytotoxicity of PVL was not attenuated by DF2593A, JPE-1375, BM213 or NQ301 (Fig. 1; Table 1).

**Table 1:** Half-maximal effective concentrations (EC_50_) of Panton-Valentine leukocidin (PVL) at different concentrations of potential competitors

Competitor	Concentration	EC_50_ PVL mean (± SD)	EC_50_ PVL fold change	EC_50_ adj. *p* value^a^	Dose-response curve adj. *p* value^a^
Avacopan	control	0.86 (± 0.30)	ref	ref	ref
	1 nM	0.79 (± 0.36)	0.92	1	0.21
	10 nM	0.67 (± 0.26)	0.78	0.96	0.05
	100 nM	0.98 (± 0.35)	1.14	0.99	0.92
	1 µM	2.07 (± 0.96)	2.40	**0.001**	**< 0.001**
	10 µM	3.14 (± 1.84)	3.64	**< 0.001**	**< 0.001**
DF2593A	control	0.59 (± 0.06)	ref	ref	ref
	1 nM	0.56 (± 0.09)	0.95	1	0.97
	10 nM	0.51 (± 0.06)	0.87	1	0.43
	100 nM	0.56 (± 0.07)	0.95	1	0.86
	1 µM	0.52 (± 0.1)	0.88	1	0.42
	10 µM	0.51 (± 0.1)	0.87	1	0.41
PMX205	control	0.5 (± 0.13)	ref	ref	ref
	1 nM	0.49 (± 0.15)	0.98	1	0.73
	10 nM	0.35 (± 0.24)	0.70	0.98	0.57
	100 nM	0.37 (± 0.3)	0.74	0.99	0.92
	1 µM	0.73 (± 0.23)	1.46	0.91	0.91
	10 µM	1.85 (± 0.49)	3.67	**< 0.001**	**< 0.001**
W-54,011	control	0.51 (± 0.06)	ref	ref	ref
	1 nM	0.43 (± 0.07)	0.85	1	0.43
	10 nM	0.43 (± 0.06)	0.85	1	0.30
	100 nM	0.43 (± 0.06)	0.84	1	0.45
	1 µM	0.83 (± 0.25)	1.63	0.74	**< 0.001**
	10 µM	2.16 (± 0.1)	4.25	**< 0.001**	**< 0.001**
BM213	control	0.58 (± 0.2)	ref	ref	ref
	1 nM	0.58 (± 0.2)	1.00	1	0.99
	10 nM	0.6 (± 0.26)	1.03	1	1
	100 nM	0.52 (± 0.18)	0.89	1	0.94
	1 µM	0.41 (± 0.08)	0.70	0.97	0.27
	10 µM	0.44 (± 0.09)	0.76	0.99	0.71
JPE-1375	control	0.48 (± 0.03)	ref	ref	ref
	1 nM	0.43 (± 0.04)	0.89	1	0.69
	10 nM	0.44 (± 0.04)	0.92	1	0.66
	100 nM	0.41 (± 0.06)	0.85	1	0.33
	1 µM	0.42 (± 0.07)	0.89	1	0.57
	10 µM	0.43 (± 0.07)	0.91	1	0.81
NQ301	control	1.12 (± 0.25)	ref	ref	ref
	1 nM	0.77 (± 0.1)	0.69	0.66	**0.01**
	10 nM	0.92 (± 0.23)	0.82	0.94	0.20
	100 nM	0.87 (± 0.19)	0.78	0.88	0.09
	1 µM	0.81 (± 0.14)	0.72	0.75	**0.02**
	10 µM	0.72 (± 0.25)	0.64	0.54	**< 0.001**

^a^Two-way ANOVA with Dunnett’s *post hoc* test for multiple comparisons

At low competitor concentrations (1–100 nM), we observed a paradoxical effect, wherein most of the compounds enhanced the cytotoxic effect of PVL (Fig. 1). Although none of the compounds showed a significant shift towards a lower EC_50_ of PVL, this paradoxical effect was strongest in NQ301 and BM213 (Fig. 1). This effect was statistically significant for NQ301 when comparing the dose-response curves at different competitor concentrations (1 nM, 1 µM, 10 µM) compared to the control (Table 1).

None of the compounds, by themselves, affected PMN viability at any concentration (Fig. S1A). Since most of the compounds were dissolved in DMSO, we tested the highest concentration of DMSO diluent used in the experiments (0.1%) and could not detect an increase in cytotoxicity compared to the PVL control (Fig. S1B).

To simulate a possible therapy option during an infection with PVL-positive *S. aureus*, we tested whether an antagonist could still attenuate the cytotoxic effect of PVL on PMNs when added after the toxin. Addition of the antagonist with the highest inhibitory potential in our study, avacopan, 30 min after PVL did not show a significant shift of the EC_50_ of PVL (Fig. S2, Table S1). Only when comparing the dose-response curves, a statistically significant difference was observed (*p* = 0.017, Table S1).

### Compounds differently affect PVL-induced IL-1β secretion

To assess the effect of the (ant)agonists on the PVL-induced cytotoxicity of monocytes and inflammasome activation, we treated human monocytes with PVL (0, 0.25, 0.5 nM) in the presence or absence of C5aR (ant)agonists (10 µM) which have been shown to be either protective or ineffective against PVL-induced cytotoxicity in PMNs (Fig. 1).

**Fig. 2:**
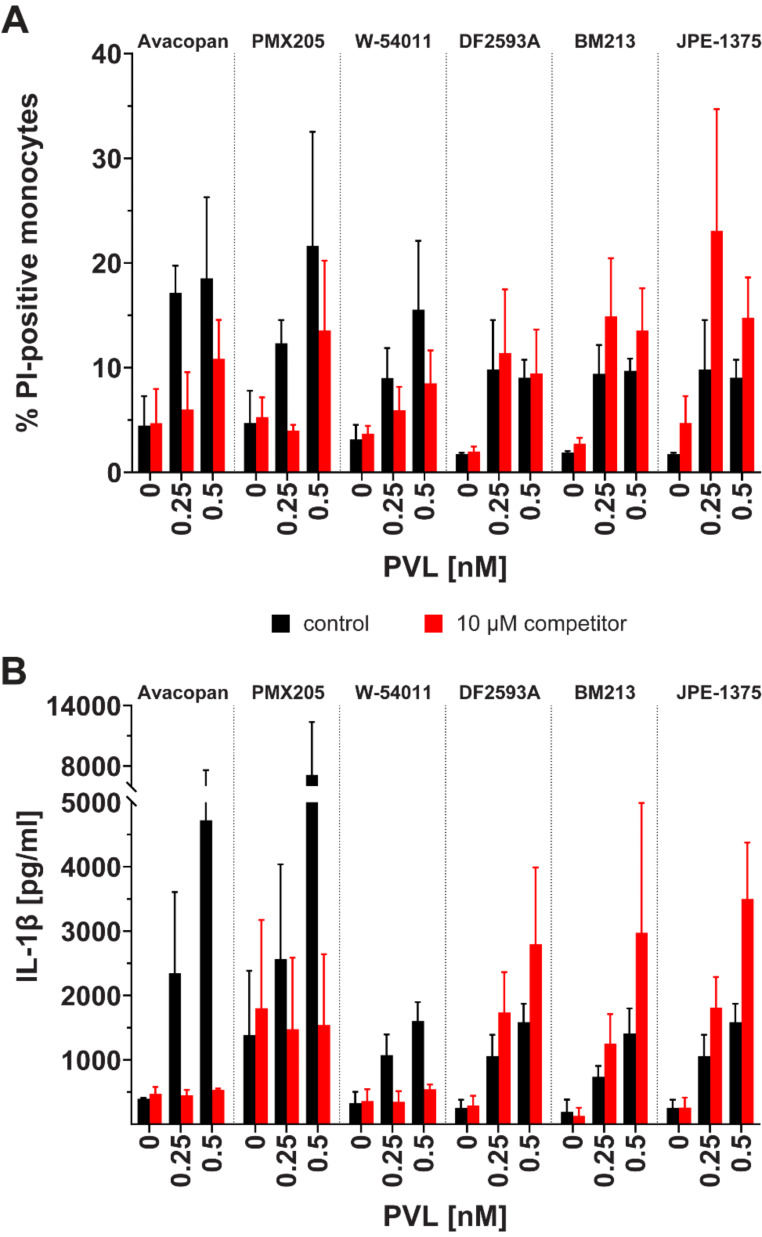
Differential effects of C5a receptor (ant)agonists on PVL-induced cytotoxicity (**A**) and IL-1β secretion (**B**) in human monocytes. Percentage of damaged monocytes after treatment with PVL (0-0.5 nM) in absence (black bars) or presence (red bars) of 10 µM competitor is displayed as percentage of PI-stained cells quantified by flow cytometry. Levels of IL-1β (in pg/ml) in culture supernatant were quantified by ELISA. Data represent mean ± SEM of three independent experiments (Tables S2 and S3)

Similar to the results obtained with PMNs, avacopan, PMX205, and W-54,011 had a protective effect on PVL-induced cytoxicity on monocytes, while DF2593A, BM213, and JPE-1375 showed no inhibition of PVL (Fig. 2A). BM213 and JPE-1375 even enhanced the cytotoxic effect on monocytes (Table S2).

Not only cytotoxicity but also inflammasome activation was impaired by avacopan, PMX205, and W-54,011 in monocytes after exposure to PVL. For instance, IL-1β secretion after treatment with 0.25 and 0.5 nM PVL was reduced when C5a receptor antagonists avacopan (5.2- and 8.9-fold), PMX205 (1.7- and 4.6-fold), or W-54,011 (3.1- and 3.0-fold) were added (Fig. 2B). In contrast, the addition of C5a receptor (ant)agonists DF2593A, BM213, or JPE-1375 enhanced IL-1β secretion at both PVL concentrations (1.8- to 2.2-fold at 0.5 nM PVL, Table S3).

## Discussion

We screened several compounds that interact with C5aR or CD45 to attenuate PVL-induced cytotoxicity and inflammasome activation in vitro.

Of the tested compounds, avacopan, PMX205 and W-54,011 were able to mitigate the PVL-induced cytotoxicity on PMNs and inflammasome activation in monocytes. Avacopan is approved for the treatment of ANCA-associated vasculitis and is contra-indicated in patients with serious infections [10]. Thus, safety issues in patients with PVL-positive *S. aureus* infections need to be addressed first before this compound can be considered as a virulence-modifying adjuvant to antimicrobial therapy in future trials. In addition, the Maximum Recommended Human Dose (MRHD) of avacopan is 349 ng/ml (0.6 µM) which is less than the lowest concentration that had a significant effect on PVL attenuation (1 µM, Fig. 1; Table 1). PMX205 is an anti-inflammatory drug currently tested in a phase 1 dose escalation trial (ACTRN12619001639112 on www.anzctr.org.au); data on the plasma pharmacokinetics after a single dose of PMX205 (0.02 mg/kg), including a dose escalation to up to 0.4 mg/kg are pending. Similarly, and to the best of our knowledge, W-54,011 has not been tested yet in clinical trials. We therefore cannot assess if the concentrations of PMX205 or W-54,011 that significantly reduced the PVL-induced cytotoxicity in PMNs can be safely achieved in humans.

We observed that low concentrations (1–100 nM) of some compounds had a paradoxical effect: PVL cytotoxicity on PMNs was slightly enhanced, particularly within the range of 0.5 to 2 nM PVL, and even for competitors with a protective effect at higher concentrations (Fig. 1). It has previously been reported that low concentrations of PVL induce apoptosis rather than necrosis [11], by binding of the LukS-PV subunit to the C5a receptor [12]. It could be speculated that binding of the (ant)agonists to C5aR influences the two modes of action of PVL on neutrophils in different ways: induction of apoptosis is stimulated while pore formation and subsequent necrosis are inhibited.

Although all C5aR (ant)agonists tested in our study (avacopan, PMX205, W-54011, DF2593A, JPE-1375, BM213) have been described as potent C5a competitors [13, 14] they showed converse effects on PVL-induced cytotoxicity on PMNs and inflammatory cytokine response in monocytes. The observed differences could be related to the specific binding affinities and mechanisms exhibited by the compounds.

Binding of the LukS subunit to the C5a receptor has been reported with a dissociation constant (K_d_) of 127 nM [3]. The binding affinity (reported as inhibition constant K_i_) of avacopan (0.1 nM) and W54011 (2.2 nM) was comparable to that of C5a (0.57 nM), whereas PMX205 (220 nM) and JPE-1375 (111 nM) were much lower [13]. Although two different measures of binding (K_d_ and K_i_) were used, they can be compared to some extent. In agreement with our results, avacopan and W54011 have a high binding affinity to C5aR, most likely higher than that of LukS. However, PMX205 has a relatively low affinity but was able to compete with PVL in our experiments. This could be explained by the different modes of interaction of the competitors with the C5aR.

The non-peptide antagonists avacopan and W-54,011 interact with an allosteric site on C5aR, formed by residues of the transmembrane domains and may thereby stabilize the inactive receptor state [15]. DF2593A is an additional non-peptide, allosteric inhibitor, but targets a different allosteric pocket of C5aR [15]. PMX205 is an analogue of PMX53, a cyclic peptidomimetic antagonist that mimics the C-terminal structure of C5a, allowing it to bind to the orthosteric site of C5aR and compete directly with the ligand [13, 15]. Similarly, JPE-1375 and BM213 target the orthosteric binding pocket of C5aR, with JPE-1375 being another but linear analogue of PMX53 and BM213 being a linear C5aR1-selective agonist [13, 16]. Despite targeting similar sites to exert their orthosteric action, only PMX205 and not JPE-1375 and BM213 antagonized PVL-induced cytotoxicity.

Variable modes of ligand-receptor interaction may differently compete for binding with PVL or stabilize C5aR1 in different conformations, interfering with subsequent pore-formation. In agreement, W-54,011 has been shown to decrease binding of PVL to C5aR and reduce its activity on neutrophils, while it potentiated that of a different bicomponent pore-forming toxin HlgC/HlgB [17].

Strikingly, the converse effect of the different C5aR (ant)agonists on PVL-induced PMN cytoxicity was even more evident in the monocyte immunomodulation assay. Avacopan, PMX205 and W-54,011 inhibited the release of pro-inflammatory IL-1β, while DF2593A, JPE-1375 and BM213 even synergistically enhanced the inflammasomal response (Fig. 2). Interestingly, JPE-1375 and BM213 previously showed immunomodulatory effects and their orthosteric competition with C5a was stronger than allosteric competition by the non-peptide antagonists avacopan and W-54,011 [13]. However, in a *Neisseria meningitidis* infection study, pharmacological blockade of C5aR by PMX205 as well as W-54,011 reduced the inflammatory cytokine release and enhanced mouse survival in a sepsis model [18].

We observed an increase in PVL toxicity when PMNs were treated with the CD45 allosteric inhibitor NQ301. It is unclear whether this is mediated by a direct effect on the interaction of CD45 with the LukF-PV subunit [4] or indirectly affecting C5aR.

In the absence of detailed structural knowledge of the binding of PVL to C5aR/CD45 at the molecular level, the reason for the differential competition with PVL displayed by various C5aR or CD45 (ant)agonists remains elusive. Nevertheless, our study demonstrates that small compounds targeting the cellular receptor of PVL provide a rationale for therapeutic intervention and the development of adjunctive treatments for PVL-related infections.

Our study has several limitations. First, the experiments were conducted with a small group of participants. Although the findings provide valuable insights, the small sample size may limit the generalizability of the results to a broader population. Given the interpersonal variance in the effect of PVL on leukocytes, we consider this limitation to be relevant. However, our approach of testing the use of C5aR antagonists against PVL-induced cytotoxicity was designed to provide a qualitative rather than a quantitative answer. Even with a small donor sample size, some compounds could be identified as beneficial or detrimental. Second, the isolated cell system used in this study may not account for potential influences of host-pathogen interactions, including other components of the immune system and conditions at sites of infection. The extent to which our in vitro results on PVL competition translate to in vivo infection with PVL-positive *S. aureus* needs to be evaluated in appropriate animal models, e.g. rabbits. Third, we did not test our recombinant PVL for LPS contamination. The PVL used in our experiments was produced in one batch and pooled, with aliquots stored at -80 °C to ensure comparability of experiments. The PVL-specific inhibition demonstrated in our study suggests that the observed effects must be C5aR specific. Lastly, our in vitro setting may not accurately reflect physiological concentrations of PVL [19] and recommended doses of inhibitor compounds in plasma. Further studies are needed to quantify the amount of PVL at sites of infection (e.g. abscesses) and whether compounds can be applied locally in adjuvant treatment.

In conclusion, C5aR-antagonizing compounds, particularly avacopan, are promising candidates to repurpose drugs in the treatment of PVL-related infections. Further in vivo experiments using suitable animal models (e.g. rabbit or humanized mouse model) are imperative to validate the efficacy of these compounds. Whether they can be repurposed as adjuvants in combination with standard antimicrobial therapies needs to be tested in clinical trials.

## Electronic supplementary material

Below is the link to the electronic supplementary material.

Supplementary Material 1

## Data Availability

Data is provided within the manuscript or supplementary information files.

## Abbreviations

PVL: Panton-Valentine leukocidin
C5aR: Complement 5a receptor
PMNs: Polymorphonuclear leukocytes
EC_50_: Half-maximal effective concentration
SEM: Standard error of mean
