# Supplementary material for: Exploration of compounds to inhibit the Panton-Valentine leukocidin of Staphylococcus aureus
Source: Med Microbiol Immunol. 2024 Sep 19;213(1):19. doi: 10.1007/s00430-024-00803-1 (PMC11413081; doi:10.1007/s00430-024-00803-1)
Supplement: Supplementary file 1 — Supplementary Material 1 [file 430_2024_803_MOESM1_ESM.pdf]

Exploration of compounds to inhibit the Panton-Valentine leukocidin of *Staphylococcus aureus*

Authors: Tobias Grebe[a,*,#], Mithra Tatjana Sarkari[a,#], Angelika Cherkaoui[a], Frieder Schaumburg[a]

Affiliations:

[a]University of Münster, Institute of Medical Microbiology, Domagkstraße 10, 48149 Münster, Germany

*Corresponding author. Adress: University of Münster, Institute of Medical Microbiology, Domagkstraße 10, 48149 Münster, Germany. Email: Tobias.Grebe@ukmuenster.de

The PDF file includes:

Fig. S1

Fig. S2

Table S1

Table S2

Table S3

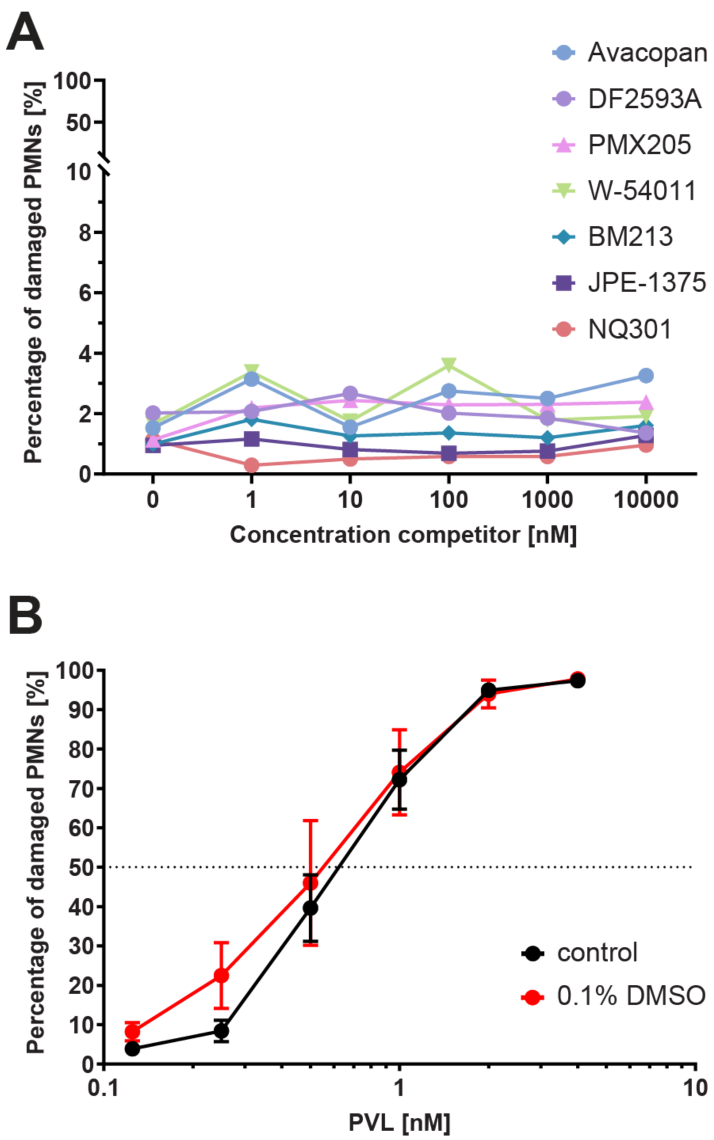

Fig. S1 Compound and diluent controls (A) C5a receptor (ant)agonists alone did not affect PMN vitality at the concentrations used in the experiments. (B) The highest concentration of DMSO (0.1%) present in the experiments did not significantly affect PVL toxicity.

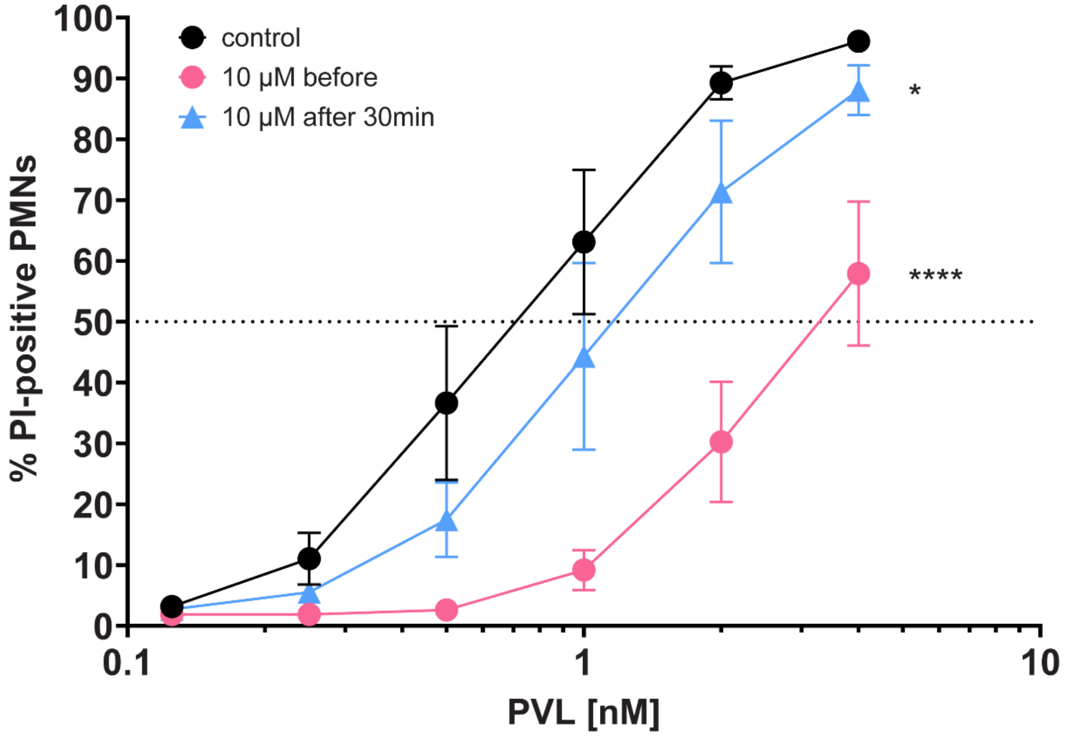

Fig. S2 Inhibition of Panton-Valentine leukocidin (PVL)-induced cytotoxicity on polymorphonuclear neutrophils (PMNs) by avacopan Inhibitor concentrations ranged from 1 nM to 10 µM. C5a receptor antagonist avacopan was added at a concentration of 10 µM to PMNs prior to or 30 minutes after incubation with PVL (0.125-4 nM). The cytotoxic effect of PVL is displayed as mean percentage ± SEM of propidium iodide (PI)-stained PMNs from three independent experiments quantified by flow cytometry. Shifts in dose-response curves were analyzed by two-way ANOVA (* p<0.05, **** p<0.0001). Results of statistical analyses are given in Table S2.

Table S1 Half-maximal effective concentrations (EC_{50}) of Panton-Valentine leukocidin (PVL) at highest concentration of C5a receptor antagonist avacopan added prior to PVL or after 30 minutes of PVL treatment.

**Competitor**	**Concentration / Condition**	**EC_{50} PVL mean (±SD)**	**EC_{50} PVL fold change**	**EC_{50} adj. *p* value[a]**	**Dose-response curve adj. *p* value[a]**
**Avacopan**	Control	0.817 (±0.415)	ref	ref	ref
	10 µM prior	4.089 (±2.262)	5.00	**0.006**	**<0.001**
	10 µM after 30 min	1.403 (±0.856)	1.72	0.743	**0.017**

[a]Two-way ANOVA with Dunnett's *post hoc* test for multiple comparisons.

Table S2 Effects of C5a receptor (ant)agonists on PVL-induced cytotoxicity in human monocytes Mean percentage (± SEM) of PI-stained monocytes after treatment with PVL (0, 0.25, 0.5 nM) in absence (control) or presence (10 µM) of the tested compounds.

		PVL concentration		
	**Condition**	**0 nM**	**0.25 nM**	**0.5 nM**
**Avacopan**	Control	4.5 (±2.8)	17.2 (±2.6)	18.5 (±7.8)
	10 µM	4.7 (±3.3)	6.0 (±3.6)	10.8 (±3.7)
**PMX205**	Control	4.7 (±3.1)	12.3 (±2.2)	21.6 (±10.9)
	10 µM	5.3 (±1.9)	4.0 (±0.6)	13.6 (±6.7)
**W-54011**	Control	3.1 (±1.4)	9.0 (±2.8)	15.5 (±6.6)
	10 µM	3.7 (±0.8)	5.9 (±2.2)	8.5 (±3.1)
**DF2593A**	Control	1.7 (±0.1)	9.8 (±4.7)	9.0 (±1.7)
	10 µM	2.0 (±0.5)	11.4 (±6.1)	9.4 (±4.2)
**BM213**	Control	1.9 (±0.2)	9.4 (±2.8)	9.7 (±1.2)
	10 µM	2.7 (±0.6)	14.9 (±5.6)	13.5 (±4.1)
**JPE-1375**	Control	1.7 (±0.1)	9.8 (±4.7)	9.0 (±1.7)
	10 µM	4.7 (±2.6)	23.1 (±11.6)	14.8 (±3.9)

Table S3 Effects of C5a receptor (ant)agonists on PVL-induced IL-1β secretion in human monocytes Mean levels (± SEM) of IL-1β in monocyte supernatant (in pg/ml) after treatment with PVL (0, 0.25, 0.5 nM) in absence (control) or presence (10 µM) of the tested compounds.

		PVL concentration		
**Competitor**	**Condition**	**0 nM**	**0.25 nM**	**0.5 nM**
**Avacopan**	Control	395 (±14)	2347 (±1259)	4720 (±2852)
	10 µM	469 (±112)	449 (±85)	531 (±25)
**PMX205**	Control	1383 (±1001)	2566 (±1471)	7112 (±5243)
	10 µM	1803 (±1370)	1475 (±1111)	1539 (±1100)
**W-54011**	Control	327 (±175)	1073 (±322)	1602 (±294)
	10 µM	360 (±185)	347 (±166)	543 (±75)
**DF2593A**	Control	255 (±127)	1056 (±334)	1585 (±287)
	10 µM	291 (±152)	1736 (±627)	2794 (±1194)
**BM213**	Control	192 (±192)	737 (±169)	1408 (±392)
	10 µM	128 (±127)	1253 (±457)	2975 (±2014)
**JPE-1375**	Control	255 (±127)	1056 (±334)	1585 (±287)
	10 µM	259 (±156)	1809 (±477)	3497 (±876)
